# Electric field and air ion exposures near high voltage overhead power lines and adult cancers: a case control study across England and Wales

**DOI:** 10.1093/ije/dyz275

**Published:** 2020-04-15

**Authors:** Mireille B Toledano, Gavin Shaddick, Kees de Hoogh, Daniela Fecht, Anna Freni Sterrantino, James Matthews, Matthew Wright, John Gulliver, Paul Elliott

**Affiliations:** 1 UK Small Area Health Statistics Unit, MRC-PHE Centre for Environment and Health, Imperial College London, London, UK; 2 National Institute for Health Research (NIHR) Health Protection Research Unit (HPRU) on Health Impact of Environmental Hazards, Imperial College London, London, UK; 3 Department of Mathematical Sciences, University of Exeter, Truro, UK; 4 Swiss Tropical and Public Health Institute, Basel, Switzerland; 5 University of Basel, Basel, Switzerland; 6 Atmospheric Chemistry Research Group, University of Bristol, Bristol, UK; 7 Centre for Environmental Health and Sustainability, University of Leicester, Leicester, UK; 8 Imperial College Biomedical Research Centre, Imperial College London, London, UK

**Keywords:** Adult cancers, electric fields, corona ions, power lines

## Abstract

**Background:**

Various mechanisms have been postulated to explain how electric fields emitted by high voltage overhead power lines, and the charged ions they produce, might be associated with possible adult cancer risk, but this has not previously been systematically explored in large scale epidemiological research.

**Methods:**

We investigated risks of adult cancers in relation to modelled air ion density (per cm^3^) within 600 m (focusing analysis on mouth, lung, respiratory), and calculated electric field within 25 m (focusing analysis on non-melanoma skin), of high voltage overhead power lines in England and Wales, 1974–2008.

**Results:**

With adjustment for age, sex, deprivation and rurality, odds ratios (OR) in the highest fifth of net air ion density (0.504–1) compared with the lowest (0–0.1879) ranged from 0.94 [95% confidence interval (CI) 0.82–1.08] for mouth cancers to 1.03 (95% CI 0.97–1.09) for respiratory system cancers, with no trends in risk. The pattern of cancer risk was similar using corona ion estimates from an alternative model proposed by others. For keratinocyte carcinoma, adjusted OR in the highest (1.06–4.11 kV/m) compared with the lowest (<0.70 kV/m) thirds of electric field strength was 1.23 (95% CI 0.65–2.34), with no trend in risk.

**Conclusions:**

Our results do not provide evidence to support hypotheses that air ion density or electric fields in the vicinity of power lines are associated with cancer risk in adults.

**Key Messages**

- Reasons for an increased cancer risk with proximity to power lines in many childhood studies remain to be elucidated.
- This large national study is the first to systematically investigate proposed hypotheses concerning effects of corona ions and electric fields produced by high voltage overhead power lines on risk of adult cancers.
- We found no evidence for an association of mouth, lung or respiratory system cancers with net air ion density nor non-melanoma skin cancers with electric field strength.
- Results do not provide evidence to support the alternative hypotheses that air ion density or electric fields, as opposed to magnetic fields, in the vicinity of high voltage overhead power lines are associated with cancer risk in adults.

## Introduction

High voltage overhead power lines are a source of both magnetic and electric fields. The International Agency for Research on Cancer (IARC) designated extremely low-frequency magnetic fields as possibly carcinogenic in humans. Electromagnetic fields are ubiquitous in today’s environment and thus, even at low levels, the population attributable risk is potentially large.

In a UK national case control study of childhood cancers, a significant dose response relationship was observed with distance from power lines,^1^ but magnetic field exposures did not appear to explain the relationship.^2^ Similarly, in the first comprehensive pooled analysis of childhood leukaemia and distance to power lines, the small increased risk for residences <50 m of 200 + kV lines was not explained by exposure to magnetic fields.^3^ Moreover, in a national case control study of adult cancers, we found no association with residential magnetic fields in proximity to high voltage overhead power lines.^4^

Reasons for an increased cancer risk with proximity to power lines in many childhood studies remain to be elucidated. Henshaw *et al*.^5^ and Fews *et al*.^6^ proposed that charged corona ions produced in the vicinity of power lines might interact with airborne particles harmful to health, and these may be carried long distances from the power lines by the wind. The attachment of corona ions to pollutant aerosol particles increases the electric charge state of the air pollutants;^7^ upon inhalation, this is hypothesized to increase the probability of deposition in the mouth and respiratory tract, leading to potential for increased risk of certain cancers, in particular mouth and respiratory cancers.^7^ Additionally, Henshaw *et al.*^5^ and Fews *et al*.^8^ proposed that high electric fields at ground level in close proximity to power lines might cause an increase in deposition of radon daughter products on the skin, leading to increased risk of keratinocyte carcinoma. To evaluate these hypotheses, Swanson *et al.*^9^ examined risk of childhood leukaemia in relation to calculated corona ion exposures along the length of power lines within 600 m of children’s residential addresses, but did not find evidence to support nor to disprove the corona ion hypothesis.^9^

To date, most epidemiological investigations concerning cancer risks from overhead power lines have focused on proximity or on the magnetic rather than the electric field component, and on children.^10–13^ Here, we carry out to our knowledge the first national case-control study of cancer incidence to evaluate these hypotheses among adults living near high voltage overhead power lines in England and Wales. Specifically, we examine: (i) risk of mouth, lung and respiratory system cancers in relation to modelled estimates of air ion density up to 600 m of power lines; and (ii) risk of keratinocyte carcinoma in relation to electric fields at ground level within 25 m of power lines.

## Methods

### Study population

We identified eligible cases and controls from the national cancer register held at the UK Small Area Health Statistics Unit, Imperial College London and maintained by the Office for National Statistics (ONS) (English data) and Welsh Cancer Intelligence and Surveillance Unit (WCISU).^14^ Case cancers in England and Wales were those of people aged 15–74 years with a diagnosis of mouth, lung and all respiratory system cancers for air ion analysis, and keratinocyte carcinomas for electric field analysis. Cases were all first primary cancers diagnosed between 1974 and 2008 and were found among people living within 600 m and 25 m of a high voltage overhead power line, respectively. Diagnostic codes of the case cancers according to the Eighth, Ninth and Tenth Revisions of the International Classification of Disease are given in Supplementary Table 1, available as Supplementary data at *IJE* online. Controls were selected from a range of cancers not considered to be associated with electromagnetic fields (see Supplementary Table 2, available as Supplementary data at *IJE* online): exclusions were malignant neoplasms of lymphatic and haematopoietic tissues including leukaemias, brain and central nervous system cancers, malignant melanoma, female breast cancer and cancers of ill-defined, secondary and unspecified sites. Cancer registrations are structured as dynamic databases: each year the records get updated if cases were missed in the previous release. The coverage for ONS is accurate at 99%; however, regional differences may still occur.^15^ Moreover, for keratinocyte carcinoma, cases tend to be underestimated and caution should be used in interpreting these cases for changes over space and time.^15^

We obtained from National Grid the geography and construction dates of the ∼21 800 pylons for all the highest voltage (400 and 275 kV) overhead power lines in England and Wales, together with the few 132 kV lines (0.1% of the total at this voltage, by length) that form part of the National Grid rather than regional distribution networks (Supplementary Figure 1, available as Supplementary data at *IJE* online). We constructed zones delineating 600 m of the power lines for each year 1969–2008 and linked these within a Geographical Information System (GIS) to the cancer database residential addresses. Residential addresses are geocoded via the Ordnance Survey ADDRESS-POINT data,^16^ representing the residential building centroid with 0.1-m accuracy. Depending on year, we successfully located 89% to 96% of the cancer database addresses.

Based on diagnosis address, we first calculated distances from power lines for year prior to diagnosis. This was done to ensure that cases and controls were living near an operational power line at time of diagnosis, since new power lines could be added to the network at any point during a year but information on the power line network was only available annually. We included all eligible cases of mouth and respiratory system cancers within 600 m and keratinocyte carcinoma within 25 m of a power line.

We identified a pool of 72 839 possible control cancers within 600 m of a power line. We randomly selected control cancers from the pool, with replacement, for the different case cancers; of the total of 47 057 control cancers thus selected, 15 174 (32.2%) were used for more than one case cancer type (see Supplementary Table 1b). We included three controls per case for mouth cancers, and one per case for the other cancers. Controls were frequency-matched to cases by year of diagnosis and region for mouth and respiratory system cancers and by year of diagnosis only for keratinocyte carcinoma (because of exhaustion of the control pool). We then obtained distances of diagnosis address from operating power lines for year of diagnosis and 5 years previously (minimum latency for solid tumours)^17^ for epidemiological analyses.

### Air ion exposures

A GIS-based model for estimating long-term (annual average) net air ion density near power lines was used to provide an (order of magnitude) approximation in this epidemiological study. Air ion densities (per cm^3^) were estimated within 600 m of power lines in England and Wales, 1974–2008, at address locations of cases and controls: 112 631 incident adult cancer cases and controls for the year of diagnosis, and 111 175 for the 5 years prior to diagnosis (the number of cases/controls was fewer for the 5-year estimates due to the construction of new power lines between the two periods). Our modelled air ion densities relate only to corona ions produced by power lines; we assume the ions then have equal chance of attachment to a homogeneous distribution of particles within 600 m of power lines. Our aim therefore was to model exposures to air ion density as a proxy for inhaled charged particle exposures related to corona ions from power lines. Our modelling approach used the following core inputs:

- geography of the power lines network between 1969 and 2008;
- wind direction patterns for each year (1969 to 2008);
- locations of receptors (i.e. x, y coordinates of residential addresses of cases and controls).

We used historical data on wind direction from a large network of meteorological sites in the UK archived at the British Atmospheric Data Centre[www.badc.ac.uk]. The coverage and completeness of these data vary in both space and time, with some sites providing continuous hourly records and others providing only partial data coverage.

We developed a model to calculate annual average air ion densities, excluding a wind speed term because of uncertainties in the relationship of wind speed to corona ion concentrations,^18^ and the short-term variability in wind speeds compared with long-term (annual) exposures estimated by our model. We also excluded a term for distance, as studies^19^^,^^20^ that looked at the relationship between air ion density and distance were limited to within 100 m of power lines, but we accounted for distance geometrically (Supplementary Figure 2, available as Supplementary data at *IJE* online).

The equation model is given below:

$$\mathrm{nAID}\left(x,y\right)=\frac{\sum_{i=1}^{12}{P_{i}f}_{S}}{\sum f_{\mathrm{tot}}}$$

where nAID (x, y) is the estimated net air ion density in arbitrary units on a continuous scale from 0 to 1 (i.e. set to a value of 1 at the source) at the receptor (i.e. residential address) defined by six-figure British National Grid coordinates (x, y); P is the value assigned to each 30˚ wind direction sector i (ranging from 1 to 12) in the GIS to denote presence (1) or absence (0) of a power line; fs is the frequency of hours for each year where wind directions fall within each 30˚ sector; ftot is the total number of wind measurements for each year (i.e. maximum is 8760 or 8784 in a leap year).

Application of this model to calculate annual mean net air ion density for hypothetical address locations is illustrated in Supplementary Figure 2. To check that our model estimated the expected air ion densities at different distances, we compared our model estimates with limited information on air ion density around high voltage power lines in the open literature. We supplemented this with data from a PhD study in Bristol,^18^ later published in Wright *et al*.,^21^ together with measurement data from an air ion monitoring campaign using portable air ion counters (AlphaLab Inc., calibrated prior to monitoring by the manufacturer) collected during the course of this study from one area in Northamptonshire. The monitoring was carried out over 12 days (18 ) between February and April 2011 at two sites in Northamptonshire, 60 m and 120 m downwind of a 400-kV power line with two sub-conductors (i.e. cables) per conductor bundle, capturing 11 h of contemporaneous data on air ion densities from fair weather conditions.

**Figure 1.**
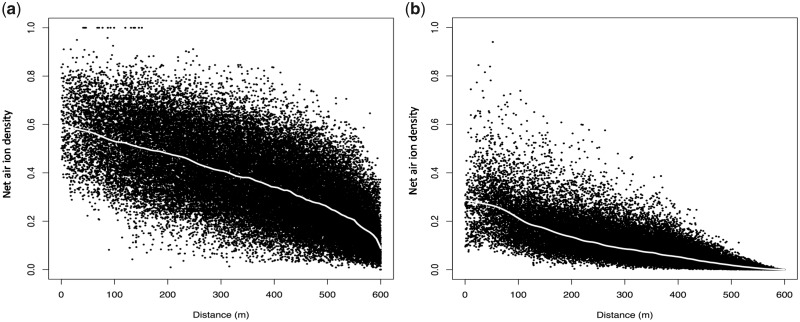
Modelled net air ion density in arbitrary units at address locations of study controls (year of diagnosis) (*n* = 63 404) by distance from the nearest high voltage power line: a) the model developed in this study, and b) implementation of the model by Swanson *et al*.^9^ The white line is the locally weighted regression (LOWESS) line.

We also implemented the Swanson model^9^ for the year of diagnosis and the preceding 5 years, to be used for sensitivity analysis in our epidemiological investigation. Although the Swanson model has a stronger distance-decay effect on net air ion density than our model (Figure 1a and b), and includes terms for wind speed and source strength, which are not included in our model, the correlation between estimates of net air ion density from the two models was high (Spearman’s rho = 0.85, *P* <0.001). This is because the main determinant of spatial variability in net air ion density associated with power lines^18^^,^^22^^,^^23^ is wind direction (i.e. upwind or downwind of power lines) which is included in both models.

### Electric fields

National Grid provided estimates of electric fields (kV/m) (blind of case/control status) for year of diagnosis for all addresses (cases/controls) lying within 25 m^24^ of an operating high voltage power line, supplemented by an additional ∼10% addresses within 25 m to increase confidentiality. To estimate the electric field, the clearance at the point closest to each address was calculated and infinite straight line conductors were assumed at this clearance. A calculated electric field estimate was obtained for 256 (71.9%) addresses, but data were insufficient (e.g. due to addresses being close to 132-kV lines, single circuit lines or lines shared with another company) to provide an estimate for the remaining 100 (28.1%) addresses.

### Information on potential confounders

In addition to stratifying by year of diagnosis/region, we included as potential confounders age, sex, small-area (enumeration district) measures of rurality, and deprivation (individual-level information on deprivation is unavailable in the cancer registry). For rurality, we used CORINE 1990/2000 land cover data^25^: codes 1–11, urban; >11, rural. A measure of rurality was considered important to include because there can be other potential sources of air ions in urban areas that are not present in rural areas. We used Carstairs score^26^ to denote deprivation, as this was available for all 35 years of the study. Negative Carstairs scores indicate areas that are more affluent, and positive scores more deprived, than the average across England and Wales. We used Carstairs 1981 at enumeration district level for cancers diagnosed 1974–85, Carstairs 1991 for 1986–95 and thereafter Carstairs 2001, based on census output areas (on average, there are approximately 12 000 enumeration districts or output areas per region).

### Statistical methods

We used logistic regression to estimate cancer risks by approximate fifths of net air ion density per cm^3^ and approximate thirds of electric field (kV/m) (based on distribution among controls), both unadjusted and adjusted for age, sex, deprivation and rurality. Since deprivation and rurality are area (enumeration district)-level measures, this leads to a multilevel data structure; we therefore included area-level random effects in our models. *P*-values for tests of linear trend across categories were based on median net air ion density per cm^3^/electric field in each category. Additional analyses treated net air ion density per cm^3^ and electric field as continuous measures in linear models. Non-linearity was tested for by including a quadratic term. We carried out analyses for year of diagnosis and also, for the air ion analyses, for 5 years previously (this latter analysis was not done for electric fields, since estimates 5 years prior to diagnosis were the same as for year of diagnosis for >95% of cases/controls). All analyses were carried out in the statistical package R.^27^

The study received ethics approval from the London MREC committee (reference: 05/MRE02/37).

## Results

There were 3061, 26 087 and 28 134 mouth, lung and respiratory system cancer registrations, respectively, included within 600 m of power lines (Table 1). Compared with case cancers, there was a higher proportion of females among controls and their address locations were more affluent and rural. Controls for mouth cancer were on average older than cases; for lung and respiratory system cancers, controls were younger than cases and had higher mean modelled net air ion density (Table 1).

**Table 1.** Descriptive statistics for year of diagnosis by modelled net air ion density per cm^3^

*n*	Mean (SD) (year of diagnosis, YOD)	Mean (SD) (5 years prior to diagnosis)	Mean (SD) (Swanson *et al*. model, YOD)	Mean (SD) (Swanson *et al*. model, 5 years prior to diagnosis)	Mean age (years)	% Female	Mean (SD) Carstairs score	% Urban
Mouth cancers								
Cases (3061)	0.342 (0.170)	0.343 (0.164)	0.077 (0.086)	0.077 (0.085)	57.9	33.4	0.4 (3.2)	83.1
Controls (9183)	0.348 (0.172)	0.348 (0.167)	0.078 (0.088)	0.079 (0.088)	61.1	40.4	−0.2 (3.0)	80.6
*P*-value	0.142	0.127	0.634	0.146	<0.001	<0.001	<0.001	0.002
Lung cancer								
Cases (26 087)	0.346 (0.172)	0.346 (0.168)	0.078 (0.087)	0.079 (0.086)	63.9	31.0	0.6 (3.1)	83.2
Controls (26 087)	0.349 (0.171)	0.349 (0.168)	0.079 (0.088)	0.080 (0.088)	60.9	42.6	0.0 (3.0)	81.6
*P-*value	0.046	0.021	0.058	0.043	<0.001	<0.001	<0.001	<0.001
Respiratory system cancers								
Cases (28 134)	0.346 (0.172)	0.346 (0.168)	0.078 (0.086)	0.079 (0.086)	63.6	30.3	0.6 (3.1)	83.2
Controls (28 134)	0.349 (0.171)	0.349 (0.168)	0.079 (0.088)	0.080 (0.088)	61.0	42.4	−0.1 (3.0)	81.1
*P*-value	0.113	0.033	0.148	0.043	<0.001	<0.001	<0.001	<0.001

Within 25 m of power lines, there were 179 cases of keratinocyte carcinoma; case cancers tended to have a higher mean estimated electric field strength and to be more affluent than controls, but there were no differences between cases and controls with respect to age, sex or rurality (Table 2).

**Table 2.** Descriptive statistics for year of diagnosis by electric field strength (kV/m)

	Mean (SD) electric field strength (kV/m)	Mean age (years)	% Female	Mean (SD) Carstairs score	% Urban
Keratinocyte carcinoma					
Cases (*n* = 179)	1.3 (1.0)	60.8	45.8	−1.6 (2.4)	83.2
Controls (*n* = 177)	1.0 (0.7)	60.6	45.8	−0.7 (3.2)	89.8
*P*-value	0.018	0.809	0.993	0.003	0.069

Risks of mouth, lung, and respiratory system cancers in relation to net air ion density per cm^3^ for year of diagnosis are shown in Table 3. In unadjusted analyses, there was a reduced risk of both lung and respiratory system cancers in the highest fifth of net air ion density, and inverse trends across quantiles of net air ion density per cm^3^. With adjustment for confounders, odds ratios (OR) for mouth, lung, and respiratory cancers in the highest fifth of net air ion density, compared with the lowest fifth, ranged from 0.94 (95% CI 0.82–1.08) for mouth cancers to 1.03 (95% CI 0.97–1.09) for respiratory cancers (Table 3), and there were no clear trends in risk with net air ion density per cm^3^. Descriptive statistics and trends in risk with net air ion density per cm^3^ at diagnosis address 5 years previously were similar to those for year of diagnosis (Table 1 and Supplementary Table 2, available as Supplementary data at *IJE* online). Quadratic terms included in the regression analyses did not improve the fit of the model. Results of sensitivity analyses conducted using corona ion estimates calculated from the model developed by Swanson *et al*.^9^ were similar to those using our own model (Table 1 and Supplementary Tables 3 and 4, available as Supplementary data at *IJE* online), with no clear trends in risk of any of the cancer groups across exposure quantiles.

**Table 3.** Cancer risk for year of diagnosis by modelled net air ion density per cm^3^

Net air ion density per cm^3^ (approx. fifths^a^**)**	Number of cases	Number of controls	Unadjusted		Adjusted^b^	
			OR	95% CI	OR	95% CI
Mouth cancers						
5	590	1837	0.92	(0.81–1.04)	0.94	(0.82–1.08)
4	594	1781	0.95	(0.84–1.08)	0.96	(0.84–1.10)
3	629	1836	0.98	(0.86–1.11)	0.99	(0.87–1.13)
2	612	1913	0.91	(0.80–1.04)	0.93	(0.81–1.06)
1	636	1813	1.00		1.00	
TOTAL	3061	9180				
*P-*value (trend, categories)			0.350		0.540	
*P*-value (trend, continuous measure)			0.143		0.272	
Lung cancer						
5	5170	5232	0.96	(0.91–1.01)	1.01	(0.95–1.07)
4	5143	5251	0.95	(0.90–1.00)	0.97	(0.92–1.03)
3	5193	5227	0.97	(0.91–1.02)	0.98	(0.93–1.04)
2	5175	5125	0.98	(0.93–1.04)	0.99	(0.94–1.05)
1	5406	5252	1.00		1.00	
TOTAL	26 087	26 087				
*P*-value (trend, categories)			0.081		0.879	
*P-*value (trend, continuous measure)			0.046		0.959	
Respiratory system cancers						
5	5590	5559	0.97	(0.92–1.02)	1.03	(0.97–1.09)
4	5564	5700	0.94	(0.89–0.99)	0.97	(0.92–1.02)
3	5581	5639	0.95	(0.91–1.01)	0.98	(0.93–1.04)
2	5595	5640	0.96	(0.91–1.01)	0.97	(0.92–1.02)
1	5804	5596	1.00		1.00	
TOTAL	28 134	28 134				
*P*-value (trend, categories)			0.235		0.520	
*P-*value (trend, continuous measure)			0.113		0.497	

a Approximate fifths of net air ion density per cm^3^: 1: 0 to 0.1879; 2: 0.188 to 0.2869; 3: 0.287 to 0.3849; 4: 0.385 to 0.503; 5: 0.504 to 1.

b Adjusted for age, sex, deprivation and rurality.

Results of the electric field analyses are shown in Table 4. In unadjusted analysis, there was a positive trend in risk of keratinocyte carcinoma with continuous measure of electric field strength and across thirds of field strength. Comparing the highest with lowest third, the odds ratio was 1.53 (95% CI 0.84–2.78). After adjustment for confounders, the excess risk in the highest third was reduced by more than half (OR = 1.23, 95% CI 0.65–2.34) and there was no clear trend with electric field strength.

**Table 4.** Cancer risk for year of diagnosis by calculated electric field (kV/m)

Electric field kV/m (approx. thirds^a^**)**	Number of cases	Number of controls	Unadjusted		Adjusted^b^	
			OR	95% CI	OR	95% CI
Keratinocyte carcinoma						
3	57	40	1.53	(0.84–2.78)	1.23	(0.65–2.34)
2	37	41	1.02	(0.55–1.90)	0.86	(0.45–1.68)
1	38	43	1.00		1.00	
TOTAL^c^	132	124				
*P-*value (trend, categories)			0.017		0.396	
*P*-value (trend, continuous measure)			0.017		0.097	

a Approximate thirds of electric field strength (kV/m): 1: <0.70; 2: 0.70–1.05; 3: 1.06–4.11.

b Adjusted for age, sex, deprivation and rurality.

c Numbers exclude cases and controls for which there were insufficient data to estimate electric field strength.

## Discussion

This large national study is the first to systematically investigate proposed hypotheses concerning effects of corona ions and electric fields produced by high voltage overhead power lines on risk of adult cancers. We found no evidence for an association of mouth, lung or respiratory system cancers with net air ion density nor of keratinocyte carcinomas with electric field strength.

To date, epidemiological studies of adult cancers and residential exposures to extremely low-frequency electromagnetic fields from overhead power lines have focused mainly on risks of leukaemia, breast, and brain and central nervous system cancers in association with the magnetic field component.^4^^,^^12^^,^^13^ The alternative hypotheses addressed here concern charged ions and direct skin deposition of particles related to electric field exposures,^5^^,^^6^^,^^8^ but these have not been extensively investigated.^13^ Statistically significant increased risks of mouth and respiratory cancers downwind of power lines have been reported in one small study in Avon, England.^28^ In addition, a small number of occupational studies of electrical workers have reported excess cancer mortality, including lung cancer,^29–31^ though these findings may reflect bias or confounding especially from smoking.^31^ More recently, the corona ion hypothesis has been investigated in relation to childhood cancers across Great Britain,^9^ but the observed pattern of childhood leukaemia rates around power lines was less well explained by corona ions than by straightforward distance. The authors concluded that their findings did not support the corona ion hypothesis as the explanation for their previously reported association between childhood leukaemia and residential proximity at birth to high voltage power lines.^1^

Electric fields produced by high voltage power lines potentially alter the concentration or transport of airborne particles by polarization of neutral particles or production of ions,^32^ although power line voltage does not appear to be a main determinant of corona ion density.^18^^,^^24^^,^^33^ The onset of corona ion discharge depends on a number of factors, including conductor characteristics (number of cables per conductor bundle and spacing, diameter), the conductor’s surface irregularity (e.g. particle deposition, protrusions, contaminants, which are in part determined by age) and the prevailing meteorology,^20^^,^^34^^,^^35^ which are important determinants of the polarity of the air ion concentration in proximity to power lines in conjunction with the phasing of the negative and positive cycles of electrons.^22^^,^^34^ Negative ions have higher electrical mobility than positive ions, meaning they may have increased chance of escape from the vicinity of the high voltage cables during the AC cycle,^21^^,^^22^ but the factors determining the net polarity of air ion concentrations are not well understood.^20^^,^^22^ Furthermore, the way in which small ions attach to pollutant aerosols is highly complex and depends on factors such as aerosol size and the existing charge state.^24^

Corona ions and the aerosols they charge can be carried considerable distances from power lines downwind.^20^^,^^22^ When inhaled, electrically charged aerosol particles have a higher probability of being deposited in the lung compared with uncharged aerosols.^24^ Increased deposition occurs by the action of mirror charge forces, providing the hypothesized mechanism by which corona ions may mediate increased exposure to particulate air pollution. In relation to other sources of air ions, higher concentrations of charged particles were reported near to a busy highway than at power line sites.^36^ This however reflected overall increased (charged + neutral) particle concentrations, and was not due to enhanced particle charge probability, which remained close to background at roadsides (in contrast to proximity to high voltage power lines where particle charge probability was significantly higher).

A second hypothesis concerns the deposition of radioactive particles onto the skin in close proximity to power lines. Radioactive decay/daughter products of naturally occurring radon gas are present in air in aerosol form. The radioactive decay process introduces natural charging of these aerosols, regardless of the presence of corona ions.^32^ The electric field acts directly on the radon daughter products to increase the amplitude of oscillation, thus increasing the probability of hitting a surface (the skin) and sticking to it. Using model heads, deposition of radon decay product aerosols under high voltage power lines outdoors increased 1.4–2.9-fold.^8^

Our study has a number of limitations. First, we fit a simple GIS model on a flat world in open countryside to estimate air ion density by distance, with a term for average wind direction to model downwind versus upwind effects.^20^^,^^22^^,^^23^ Lacking information to derive a term for distance, we accounted for distance by the geometrical relationship of power lines and wind direction sectors. As distance between a power line and address location increases, there is a tendency for fewer wind direction sectors to intersect with a power line, thus reducing the number of hours per year when address locations are downwind of power lines.

Second, the factors determining the net polarity of air ions at different downwind distances are not well understood,^20^^,^^22^ nor is the relationship between the earth’s electric field, small and large ions, and ion-related particle charge numbers in the wake of the power line; these parameters have so far been simultaneously characterized in only one study.^20^ Furthermore, there is large variation in air ion density at individual measurement sites close to power lines, as well as large differences between different measurement sites located in the same region.^18^^,^^21^^,^^22^ Thus, our model may have under- or overestimated corona ion density with distance for different power line characteristics and voltage in different locations, which may have led to exposure misclassification and a weakening of any relationship between modelled air ion density and cancer risk. Similarly, the calculations made to obtain electric fields were somewhat limited in that they were based on the x, y coordinate of the case/control address only, and did not include averaging over the spatial extent of a home. They also took no account of perturbation to the field by buildings or other objects.

Third, we had no direct information on migration of cases or controls, and so were only able to estimate corona ions or electric fields at a residential address at time of diagnosis; similarly, we were unable to allow for cumulative exposures or latency, except for providing estimates at diagnosis address 5 years previously.

Fourth, we did not have available information on smoking and therefore adjusted for deprivation as a close proxy instead.^37^ Areas near power lines are more affluent than the average for England and Wales.^4^ Reflecting a positive association of lung and respiratory system cancers with deprivation,^38^^,^^39^ unadjusted analyses showed inverse associations with modelled air ion density. After adjustment for deprivation and other confounders, odds ratios moved close to one. Conversely, as keratinocyte carcinoma is associated with affluence,^40^^,^^41^ an excess risk was found with increasing electric field strength, which reduced by half after adjustment for confounders, suggesting possible residual confounding. The excess risk of 23% observed in the highest third of electric fields is consistent with the hypothesized risk,^24^ though the study was insufficiently powered to detect it reliably, even with 35 years of observation. Finally, we used as controls a range of cancers not considered to be associated with electromagnetic fields. Use of cancer controls might introduce bias (toward the null) if exposures are positively associated with the control cancers.

In conclusion, our results do not provide evidence to support the alternative hypotheses that air ion density or electric fields, as opposed to magnetic fields, in the vicinity of high voltage overhead power lines are associated with cancer risk in adults.

## Funding

This work was supported by the Department of Health, grant number RRX106. The Energy Networks Association also contributed funding through a grant to the Department of Health. A Steering Committee comprising independent experts and representatives of the funders advised on study design and commented on the protocol. Neither the Department of Health, the Energy Networks Association nor the National Grid were involved in the writing or interpretation of this report which is the responsibility of the authors alone. The UK Small Area Health Statistics Unit is funded by Public Health England (PHE) as part of the Medical Research Council Centre for Environment and Health at Imperial College London (MR/L01341X/1). We acknowledge support from the National Institute for Health Research (NIHR) Health Protection Research Unit (HPRU) on Health Impact of Environmental Hazards (HPRU-2012–10141), and (to P.E.) from the National Institute for Health Research (NIHR) Imperial Biomedical Research Centre (RDF03).

## Supplementary Material

dyz275_Supplementary_Data
