# Supplementary material for: Electric field and air ion exposures near high voltage overhead power lines and adult cancers: a case control study across England and Wales
Source: Int J Epidemiol. 2020 Apr 15;49(Suppl 1):i57–66. doi: 10.1093/ije/dyz275 (PMC7158064; doi:10.1093/ije/dyz275)
Supplement: dyz275_Supplementary_Data [file dyz275_supplementary_data.zip › dyz275-Suppl_Data/ije-2019-06-0783-File004.docx]

**Supplementary Table 1. International classification of disease (ICD) codes of: (a) Case cancers; (b) Control cancers and numbers included by case cancer type, according to eighth, ninth and tenth revisions of the ICD**

| (a) |  |  |  |
| --- | --- | --- | --- |
| **Cancer** | **ICD-10 codes** | **ICD-9 codes** | **ICD-8 codes** |
| **Mouth Cancers** | C00 - Malignant neoplasm of lip  C14 - Malignant neoplasm of other and ill-defined sites in the lip, oral cavity and pharynx | 140 - Malignant neoplasm of lip  149 - Malignant neoplasm of other and ill-defined sites within the lip, oral cavity, and pharynx | 140 - Malignant neoplasm of lip  149 - Malignant neoplasm of pharynx, unspecified |
| **Lung cancer** | C34 - Malignant neoplasm of bronchus and lung | 162 - Malignant neoplasm of trachea, bronchus, and lung | 162 - Malignant neoplasm of trachea, bronchus and lung |
| **Respiratory cancers** | C30 - Malignant neoplasm of nasal cavity and middle ear  C34 - Malignant neoplasm of bronchus and lung  C37 - Malignant neoplasm of thymus | 160 - Malignant neoplasm of nasal cavities, middle ear, and accessory sinuses  162 - Malignant neoplasm of trachea, bronchus, and lung  164.0 - Malignant neoplasm of thymus | 160- Malignant neoplasm of nose, nasal cavities, middle ear and accessory sinuses  162 - Malignant neoplasm of trachea, bronchus and lung  194.2 - Malignant neoplasm of other endocrine glands (Thymus) |
| **Keratinocyte carcinoma** | C44 Other malignant neoplasms of skin | 173 Other malignant neoplasm of skin | 173 Other malignant neoplasm of skin |

**Supplementary Table 1. continued**

| (b) |  |  |  |  | | | |
| --- | --- | --- | --- | --- | --- | --- | --- |
| **Cancer** | **ICD-10 codes** | **ICD-9 codes** | **ICD-8 codes** | **Numbers included as controls in analyses of:** | | | |
|  |  |  |  | **Mouth** | **Lung** | **Respiratory** | **NMSC*** |
| Prostate | C61 Malignant neoplasm of prostate | 185 Malignant neoplasm of prostate | 185 Malignant neoplasm of prostate | 1430 (15.57%) | 3386 (12.98%) | 3684 (13.09%) | 31 (17.51%) |
| Colon | C18 Colon | 153 Malignant neoplasm of colon | 153 Malignant neoplasm of large intestine, except rectum | 1317 (14.34%) | 3777 (14.48%) | 4072 (14.47%) | 27 (15.25%) |
| Female genital organs (excl corpus uteri) | C51 Malignant neoplasm of vulva | 179 Malignant neoplasm of uterus, part unspecified | 180 Malignant neoplasm of cervix uteri | 1081 (11.77%) | 3306 (12.67%) | 3620 (12.87%) | 23 (12.99%) |
|  | C52 Malignant neoplasm of vagina | 180 Malignant neoplasm of cervix uteri |  |  |  |  |  |
|  | C53 Malignant neoplasm of cervix uteri | 181 Malignant neoplasm of placenta | 181  Chorionepithelioma |  |  |  |  |
|  | C55 Malignant neoplasm of uterus, part unspecified | 183 Malignant neoplasm of ovary and other uterine adnexa | 183 Malignant neoplasm of ovary, fallopian tube, and broad ligament |  |  |  |  |
|  | C56 Malignant neoplasm of ovary | 184 Malignant neoplasm of other and unspecified female genital organs | 184 Malignant neoplasm of other and unspecified female genital organs |  |  |  |  |
|  | C57 Malignant neoplasm of other and unspecified female genital organs |  |  |  |  |  |  |
|  | C58 Malignant neoplasm of placenta |  |  |  |  |  |  |
| Rectum, rectosigmoid joint and anus | C19 Malignant neoplasm of rectosigmoid junction | 154 Malignant neoplasm of rectum, rectosigmoid junction, and anus | 154 Malignant neoplasm of rectum and rectosigmoid junction | 953 (10.38%) | 2698 (10.34%) | 2891 (10.28%) | 20 (11.3%) |
|  | C20 Malignant neoplasm of rectum |  |  |  |  |  |  |
|  | C21 Malignant neoplasm of anus and anal canal |  |  |  |  |  |  |
| Urinary organs excl kidney | C65-C68 Bladder and urinary organs, excl kidney | 188 Malignant neoplasm of bladder 189.1 Renal pelvis | 188 Malignant neoplasm of bladder | 865 (9.42%) | 2531 (9.7%) | 2794 (9.93%) | 10 (5.65%) |
|  |  | 189.2 Ureter | 189.1 Pelvis of kidney |  |  |  |  |
|  |  | 189.3 Urethra | 189.2 Ureter |  |  |  |  |
|  |  | 189.4 Paraurethral glands | 189.9 Other and unspecified |  |  |  |  |
|  |  | 189.8 Other specified sites of urinary organs |  |  |  |  |  |
|  |  | 189.9 Urinary organ, site unspecified |  |  |  |  |  |
| Stomach | C16 Malignant neoplasm of stomach | 151 Malignant neoplasm of stomach | 151 Malignant neoplasm of stomach | 794 (8.65%) | 2521 (9.66%) | 2629 (9.34%) | 16 (9.04%) |
| Pancreas | C25 Malignant neoplasm of pancreas | 157 Malignant neoplasm of pancreas | 157 Malignant neoplasm of pancreas | 471 (5.13%) | 1446 (5.54%) | 1579 (5.61%) | 8 (4.52%) |
| Corpus uteri | 182 Malignant neoplasm of body of uterus | 182 Other malignant neoplasm of uterus | C54 Corpus uteri | 432 (4.7%) | 1259 (4.83%) | 1311 (4.66%) | 8 (4.52%) |
| Oesophagus | C15 Malignant neoplasm of oesophagus | 150 Malignant neoplasm of oesophagus | 150 Malignant neoplasms of oesophagus | 430 (4.68%) | 1144 (4.39%) | 1211 (4.3%) | 11 (6.21%) |
| Kidney | C64 Malignant neoplasm of kidney, except renal pelvis | 189.0 Kidney, except pelvis | 189.0 Kidney, except pelvis | 394 (4.29%) | 1076 (4.12%) | 1217 (4.33%) | 9 (5.08%) |
| Biliary passages and liver | C22 Malignant neoplasm of liver and intrahepatic bile ducts | 155 Malignant neoplasm of liver and intrahepatic bile ducts | 155 Malignant neoplasm of liver and intrahepatic bile ducts, specified as primary | 211 (2.3%) | 567 (2.17%) | 566 (2.01%) | 2 (1.13%) |
|  | C24 Malignant neoplasm of other and unspecified parts of biliary tract | 156.1 Extrahepatic bile ducts | 156.1 Extrahepatic bile ducts |  |  |  |  |
|  |  | 156.2 Ampulla of Vater | 156.2 Ampulla of Vater |  |  |  |  |
|  |  | 156.8 Other specified sites of gallbladder and extrahepatic bile ducts |  |  |  |  |  |
|  |  | 156.9 Biliary tract, part unspecified | 156.9 Biliary tract, part unspecified |  |  |  |  |
| Mesothelioma, Kaposi’s sarcoma, peripheral nerves, peritoneum, connective  and soft tissues | C45-C49 Malignant neoplasms of mesothelial and soft tissue | 158 Malignant neoplasm of retroperitoneum and peritoneum | 158 Malignant neoplasm of peritoneum and retroperitoneal tissue | 202 (2.2%) | 546 (2.09%) | 652 (2.32%) | 2 (1.13%) |
|  |  | 171 Malignant neoplasm of connective and other soft tissue | 171 Malignant neoplasm of connective and other soft tissue |  |  |  |  |
|  |  | Kaposi’s sarcoma: | 192.4 Peripheral nerves |  |  |  |  |
|  |  | 176.0 Skin | 192.5 Sympathetic nervous system |  |  |  |  |
|  |  | 176.1 Soft tissue |  |  |  |  |  |
|  |  | 176.2 Palate |  |  |  |  |  |
|  |  | 176.5 Lymph nodes |  |  |  |  |  |
|  |  | 176.8 Other specified sites |  |  |  |  |  |
|  |  | 176.9 Unspecified |  |  |  |  |  |
| Testicular cancer | C62 Malignant neoplasm of testis | 186 Malignant neoplasm of testis | 186 Malignant neoplasm of testis | 201 (2.19%) | 524 (2.01%) | 557 (1.98%) | 2 (1.13%) |
| Thyroid gland | C73 Malignant neoplasm of thyroid gland | 193 Malignant neoplasm of thyroid gland | 193 Malignant neoplasm of thyroid gland | 111 (1.21%) | 338 (1.3%) | 350 (1.24%) | 1 (0.56%) |
| Heart, mediastinum and pleura | C38 Malignant neoplasm of heart, mediastinum and pleura | 163 Malignant neoplasm of pleura | 163 Malignant neoplasm of other and unspecified respiratory organs | 59 (0.64%) | 224 (0.86%) | 203 (0.72%) | 2 (1.13%) |
|  |  | 164.1 Heart |  |  |  |  |  |
|  |  | 164.2 Anterior mediastinum |  |  |  |  |  |
|  |  | 164.3 Posterior mediastinum |  |  |  |  |  |
|  |  | 164.8 Other |  |  |  |  |  |
|  |  | 164.9 Mediastinum, part unspecified |  |  |  |  |  |
| Small intestine | C17 Malignant neoplasm of small intestine | 152 Malignant neoplasm of small intestine, including duodenum | 152 Malignant neoplasm of small intestine, including duodenum | 54 (0.59%) | 117 (0.45%) | 121 (0.43%) | 1 (0.56%) |
| Eye | C69 Malignant neoplasm of eye and adnexa | 190 Malignant neoplasm of eye | 190 Malignant neoplasm of eye | 34 (0.37%) | 113 (0.43%) | 127 (0.45%) | 1 (0.56%) |
| Bones and articular cartilage | C40-C41 Malignant neoplasms of bone and articular cartilage | 170 Malignant neoplasm of bone and articular cartilage | 170 Malignant neoplasm of bone | 33 (0.36%) | 142 (0.54%) | 141 (0.5%) | 1 (0.56%) |
| Gallbladder | C23 Malignant neoplasm of gallbladder | 156.0 Gallbladder | 156.0 Gallbladder | 33 (0.36%) | 126 (0.48%) | 132 (0.47%) | 0 (0%) |
| Other digestive organs, inc spleen | C26 Malignant neoplasm of other and ill-defined digestive organs | 159 Malignant neoplasm of other and ill-defined sites within the digestive organs and peritoneum | 159 Malignant neoplasm of unspecified digestive organs | 29 (0.32%) | 82 (0.31%) | 87 (0.31%) | 1 (0.56%) |
| Male genital organs (excl testis and prostate) | C60 Malignant neoplasm of penis | 187 Malignant neoplasm of penis and other male genital organs | 187 Malignant neoplasm of other and unspecified male genital organs | 28 (0.3%) | 105 (0.4%) | 114 (0.41%) | 1 (0.56%) |
|  | C63 Malignant neoplasm of other and unspecified male genital organs |  |  |  |  |  |  |
| Glands (excl pituitary and thyroid) | C74 Malignant neoplasm of adrenal gland | 194.0 Adrenal gland | 194.0 Suprarenal gland | 19 (0.21%) | 49 (0.19%) | 61 (0.22%) | 0 (0%) |
|  | C75.0 Parathyroid gland | 194.1 Parathyroid gland | 194.1 Parathyroid gland |  |  |  |  |
|  | C75.2.Craniopharyngeal duct | 194.4 Pineal gland | 194.4 Pineal gland |  |  |  |  |
|  | C75.3 Pineal gland | 194.5 Carotid body |  |  |  |  |  |
|  | C75.4 Carotid body | 194.6 Aortic body and other paraganglia |  |  |  |  |  |
|  | C75.5 Aortic body and other paraganglia | 194.8 Other | 194.8 Other |  |  |  |  |
|  | C75.8 Pluriglandular involvement, unspecified | 194.9 Endocrine gland, site unspecified | 194.9 Endocrine gland, site unspecified |  |  |  |  |
|  | C75.9 Endocrine gland, unspecified |  |  |  |  |  |  |
| Pituitary gland | C75.1 Pituitary gland | 194.3 Pituitary gland and craniopharyngeal duct | 194.3 Pituitary gland and craniopharyngeal duct | 1 (0.01%) | 7 (0.03%) | 14 (0.05%) | 0 (0%) |
| Other respiratory system organs | C39 Malignant neoplasm of other and ill-defined sites in the respiratory system and intrathoracic organs | 165 Malignant neoplasm of other and ill-defined sites within the respiratory system and intrathoracic organs | 163 Malignant neoplasm of other and unspecified respiratory organs | 1 (0.01%) | 3 (0.01%) | 1 (0%) | 0 (0%) |
| **TOTAL** | | | | 9183 | 26087 | 28134 | 177 |

*NMSC – keratinocyte carcinoma

**Supplementary Table 2. Modelled net air ion density per cm^3^, 5-years prior to diagnosis.**

| **Net air ion density per cm^3^ (approx. fifths^1^)** | **Number**  **of Cases** | **Number of**  **Controls** | **Unadjusted** | | **Adjusted^2^** | |
| --- | --- | --- | --- | --- | --- | --- |
|  |  |  | **OR** | **95% CI** | **OR** | **95% CI** |
| ***Mouth cancers*** | | | | | | |
| **5** | 555 | 1813 | 0.90 | (0.79 – 1.03) | 0.93 | (0.81 – 1.07) |
| **4** | 615 | 1763 | 1.03 | (0.91 – 1.17) | 1.03 | (0.90 – 1.18) |
| **3** | 627 | 1851 | 1.00 | (0.88 – 1.14) | 1.03 | (0.90 – 1.17) |
| **2** | 619 | 1840 | 0.99 | (0.87 – 1.13) | 1.01 | (0.88 - 1.15) |
| **1** | 611 | 1806 | 1.00 |  | 1.00 |  |
| **TOTAL** | 3027 | 9073 |  |  |  |  |
| *P-value (trend, categories)* | | | 0.236 |  | 0.456 |  |
| *P-value (trend, continuous measure)* | | | 0.130 |  | 0.271 |  |
| ***Lung cancer*** | | | | | | |
| **5** | 4989 | 5101 | 0.95 | (0.90 – 1.01) | 1.00 | (0.95 – 1.06) |
| **4** | 5105 | 5201 | 0.96 | (0.91 – 1.01) | 0.99 | (0.93 – 1.05) |
| **3** | 5108 | 5096 | 0.98 | (0.92 - 1.03) | 1.00 | (0.95 – 1.06) |
| **2** | 5130 | 5077 | 0.98 | (0.93 - 1.04) | 1.00 | (0.94 – 1.06) |
| **1** | 5314 | 5177 | 1.00 |  | 1.00 |  |
| **TOTAL** | 25646 | 25652 |  |  |  |  |
| *P-value (trend, categories)* | | | 0.046 |  | 0.868 |  |
| *P-value (trend, continuous measure)* | | | 0.021 |  | 0.752 |  |
| ***Respiratory system cancers*** | | | | | | |
| **5** | 5395 | 5511 | 0.96 | (0.91 – 1.01) | 1.02 | (0.97 – 1.08) |
| **4** | 5504 | 5552 | 0.97 | (0.92 – 1.02) | 1.01 | (0.96 – 1.07) |
| **3** | 5553 | 5566 | 0.98 | (0.93 – 1.03) | 1.01 | (0.95 – 1.06) |
| **2** | 5516 | 5477 | 0.99 | (0.94 – 1.04) | 1.01 | (0.95 - 1.06) |
| **1** | 5695 | 5576 | 1.00 |  | 1.00 |  |
| **TOTAL** | 27663 | 27682 |  |  |  |  |
| *P-value (trend, categories)* | | | 0.094 |  | 0.600 |  |
| *P-value (trend, continuous measure)* | | | 0.033 |  | 0.785 |  |

^1^Approximate fifths of net air ion density per cm^3^ : 1: 0 - 0.1929, 2: 0.193 to 0.2889 3: 0.289-0.3869 , 4: 0.387-0.4999, 5: 0.500 – 1

^2^Adjusted for age, sex, deprivation and rurality

**Supplementary Table 3. Modelled net air ion density per cm^3^, Swanson et al (2014) model, year of diagnosis.**

| **Net air ion density per cm^3^ (approx. fifths^1^)** | **Number**  **of Cases** | **Number of**  **Controls** | **Unadjusted** | | **Adjusted^2^** | |
| --- | --- | --- | --- | --- | --- | --- |
|  |  |  | **OR** | **95% CI** | **OR** | **95% CI** |
| ***Mouth cancers*** | | | | | | |
| **5** | 595 | 1800 | 0.96 | (0.85 – 1.10) | 0.98 | (0.85 – 1.12) |
| **4** | 618 | 1845 | 0.98 | (0.86 – 1.11) | 0.98 | (0.86 – 1.12) |
| **3** | 590 | 1838 | 0.94 | (0.82 – 1.07) | 0.93 | (0.81 – 1.06) |
| **2** | 633 | 1878 | 0.98 | (0.86 – 1.12) | 0.98 | (0.86 - 1.12) |
| **1** | 625 | 1822 | 1.00 |  | 1.00 |  |
| **TOTAL** | 3061 | 9183 |  |  |  |  |
| *P-value (trend, categories)* | | | 0.710 |  | 0.683 |  |
| *P-value (trend, continuous measure)* | | | 0.636 |  | 0.885 |  |
| ***Lung cancer*** | | | | | | |
| **5** | 5122 | 5256 | 0.95 | (0.90 – 1.01) | 1.01 | (0.96 – 1.07) |
| **4** | 5200 | 5241 | 0.97 | (0.92 – 1.03) | 0.99 | (0.94 – 1.05) |
| **3** | 5251 | 5196 | 0.99 | (0.94 - 1.05) | 0.99 | (0.94 – 1.05) |
| **2** | 5261 | 5246 | 0.98 | (0.93 - 1.04) | 0.98 | (0.93 – 1.04) |
| **1** | 5253 | 5147 | 1.00 |  | 1.00 |  |
| **TOTAL** | 26087 | 26087 |  |  |  |  |
| *P-value (trend, categories)* | | | 0.087 |  | 0.608 |  |
| *P-value (trend, continuous measure)* | | | 0.058 |  | 0.331 |  |
| ***Respiratory system cancers*** | | | | | | |
| **5** | 5542 | 5641 | 0.99 | (0.94 – 1.05) | 1.06 | (1.00 – 1.12) |
| **4** | 5600 | 5645 | 1.00 | (0.95 – 1.06) | 1.03 | (0.97 – 1.09) |
| **3** | 5670 | 5587 | 1.03 | (0.97 – 1.08) | 1.03 | (0.98 – 1.09) |
| **2** | 5690 | 5572 | 1.03 | (0.98 – 1.09) | 1.03 | (0.98 - 1.09) |
| **1** | 5632 | 5689 | 1.00 |  | 1.00 |  |
| **TOTAL** | 28134 | 28134 |  |  |  |  |
| *P-value (trend, categories)* | | | 0.282 |  | 0.092 |  |
| *P-value (trend, continuous measure)* | | | 0.148 |  | 0.089 |  |

^1^Approximate fifths of net air ion density per cm^3^ : 1: 0 – 0.0089, 2: 0.009 to 0.0319 3: 0.032-0.0709 , 4: 0.071-0.1369, 5: 0.137 - 1

^2^ Adjusted for age, sex, deprivation and rurality

**Supplementary Table 4. Modelled net air ion density per cm^3^, Swanson et al (2014) model, 5 years prior to diagnosis.**

| **Net air ion density per cm^3^ (approx. fifths^1^)** | **Number**  **of Cases** | **Number of**  **Controls** | **Unadjusted** | | **Adjusted^2^** | |
| --- | --- | --- | --- | --- | --- | --- |
|  |  |  | **OR** | **95% CI** | **OR** | **95% CI** |
| ***Mouth cancers*** | | | | | | |
| **5** | 556 | 1776 | 0.92 | (0.81 – 1.05) | 0.94 | (0.82 – 1.08) |
| **4** | 627 | 1804 | 1.02 | (0.90 – 1.16) | 1.03 | (0.90 – 1.18) |
| **3** | 604 | 1850 | 0.96 | (0.84 – 1.09) | 0.96 | (0.84 – 1.09) |
| **2** | 619 | 1815 | 1.00 | (0.88 – 1.14) | 1.01 | (0.88 - 1.15) |
| **1** | 621 | 1828 | 1.00 |  | 1.00 |  |
| **TOTAL** | 3027 | 9073 |  |  |  |  |
| *P-value (trend, categories)* | | | 0.250 |  | 0.528 |  |
| *P-value (trend, continuous measure)* | | | 0.151 |  | 0.312 |  |
| ***Lung cancer*** | | | | | | |
| **5** | 5001 | 5159 | 0.94 | (0.89 – 1.00) | 1.00 | (0.95 – 1.06) |
| **4** | 5135 | 5179 | 0.97 | (0.91 – 1.02) | 0.99 | (0.93 – 1.04) |
| **3** | 5108 | 5112 | 0.97 | (0.92 - 1.03) | 0.98 | (0.92 – 1.04) |
| **2** | 5200 | 5136 | 0.99 | (0.93 - 1.04) | 0.99 | (0.93 – 1.04) |
| **1** | 5202 | 5066 | 1.00 |  | 1.00 |  |
| **TOTAL** | 25656 | 25625 |  |  |  |  |
| *P-value (trend, categories)* | | | 0.033 |  | 0.932 |  |
| *P-value (trend, continuous measure)* | | | 0.043 |  | 0.372 |  |
| ***Respiratory system cancers*** | | | | | | |
| **5** | 5391 | 5598 | 0.97 | (0.92 – 1.02) | 1.04 | (0.98 – 1.10) |
| **4** | 5550 | 5499 | 1.01 | (0.96 – 1.07) | 1.04 | (0.98 – 1.10) |
| **3** | 5516 | 5476 | 1.01 | (0.96 – 1.07) | 1.02 | (0.97 – 1.08) |
| **2** | 5610 | 5490 | 1.03 | (0.97 – 1.08) | 1.03 | (0.97 - 1.08) |
| **1** | 5596 | 5619 | 1.00 |  | 1.00 |  |
| **TOTAL** | 27663 | 27682 |  |  |  |  |
| *P-value (trend, categories)* | | | 0.068 |  | 0.237 |  |
| *P-value (trend, continuous measure)* | | | 0.043 |  | 0.241 |  |

^1^Approximate fifths of net air ion density per cm^3^ : 1: 0 – 0.0089, 2: 0.009 to 0.0329 3: 0.033-0.0729 , 4: 0.073-0.1389, 5: 0.139 - 1

^2^ Adjusted for age, sex, deprivation and rurality

**Supplementary Table 5. Descriptive Statistic by exposure quintiles and year of diagnosis.**

|  |  |  | **Net air ion density per cm^3^ (approx. fifths^1^)** | | | | |
| --- | --- | --- | --- | --- | --- | --- | --- |
|  |  |  | **1** | **2** | **3** | **4** | **5** |
| Outcome | Year of diagnosis |  |  |  |  |  |  |
| Mouth Cancers | 1 | Mean age (SD) | 60.53  (11.21) | 60.44 (11.03) | 60.64  (10.87) | 60.07  (11.47) | 59.70  (11.21) |
|  |  | Mean Carstairs Score (SD) | 0.08(3.05) | 0.02(3.02) | -0.01(3.02) | -0.17(2.95) | -0.26(3.11) |
|  |  | % Females | 39.66 | 39.24 | 35.64 | 39.11 | 39.69 |
|  |  | % Urban | 86.14 | 82.63 | 81.22 | 80.01 | 76.01 |
|  | 5 | Mean age (SD) | 60.39(11.17) | 60.59(11.02) | 60.56(10.89) | 60.27(11.23) | 59.53(11.45) |
|  |  | Mean Carstairs Score (SD) | 0.07(3.01) | -0.04(3.01) | -0.01(3.04) | -0.25(2.91) | -0.38(3.02) |
|  |  | % Females | 39.88 | 38.71 | 36.15 | 38.79 | 39.49 |
|  |  | % Urban | 85.18 | 81.86 | 79.17 | 79.42 | 74.68 |
| Respiratory system cancers | 1 | Mean age (SD) | 62.29(9.90) | 62.50(9.73) | 62.34(9.69) | 62.34(9.79) | 61.99(10.11) |
|  |  | Mean Carstairs Score (SD) | 0.51(3.05) | 0.37(3.04) | 0.32(3.02) | 0.18(3.02) | 0.00(3.17) |
|  |  | % Females | 36.85 | 36.31 | 36.50 | 35.83 | 36.20 |
|  |  | % Urban | 85.75 | 83.23 | 82.97 | 80.66 | 77.97 |
|  | 5 | Mean age (SD) | 62.33(9.96) | 62.41(9.73) | 62.50(9.48) | 62.23(9.90) | 62.03(10.11) |
|  |  | Mean Carstairs Score (SD) | 0.45(3.03) | 0.29(3.01) | 0.20(2.96) | 0.12(3.00) | -0.09(3.09) |
|  |  | % Females | 36.98 | 36.37 | 35.89 | 36.79 | 35.71 |
|  |  | % Urban | 84.95 | 82.46 | 81.61 | 79.92 | 77.13 |
| Lung cancer | 1 | Mean age (SD) | 62.39(9.87) | 62.42(9.88) | 62.47(9.59) | 62.51(9.65) | 62.09(10.01) |
|  |  | Mean Carstairs Score (SD) | 0.53(3.06) | 0.37(3.02) | 0.32(3.02) | 0.22(3.03) | 0.03(3.16) |
|  |  | % Females | 37.07 | 36.46 | 36.30 | 37.04 | 37.15 |
|  |  | % Urban | 85.96 | 82.82 | 83.54 | 81.55 | 78.06 |
|  | 5 | Mean age (SD) | 62.38(9.92) | 62.54(9.68) | 62.45(9.66) | 62.46(9.65) | 62.12(10.01) |
|  |  | Mean Carstairs Score (SD) | 0.47(3.04) | 0.31(2.99) | 0.22(2.97) | 0.15(3.01) | -0.07(3.07) |
|  |  | % Females | 37.11 | 36.26 | 36.17 | 37.88 | 36.65 |
|  |  | %Urban | 85.06 | 82.32 | 82.06 | 80.55 | 77.41 |

^1^ Approximate fifths of net air ion density per cm^3^: 1: 0 - 0.1879, 2: 0.188 - 0.2869, 3: 0.287 - 0.3849, 4: 0.385 - 0.5039, 5: 0.504 - 1
