# Supplementary material for: Electric field and air ion exposures near high voltage overhead power lines and adult cancers: a case control study across England and Wales
Source: Int J Epidemiol. 2020 Apr 15;49(Suppl 1):i57–66. doi: 10.1093/ije/dyz275 (PMC7158064; doi:10.1093/ije/dyz275)

**Supplementary Figure 1. Changes in power line geography from 1969 to 2008 in England and Wales.**


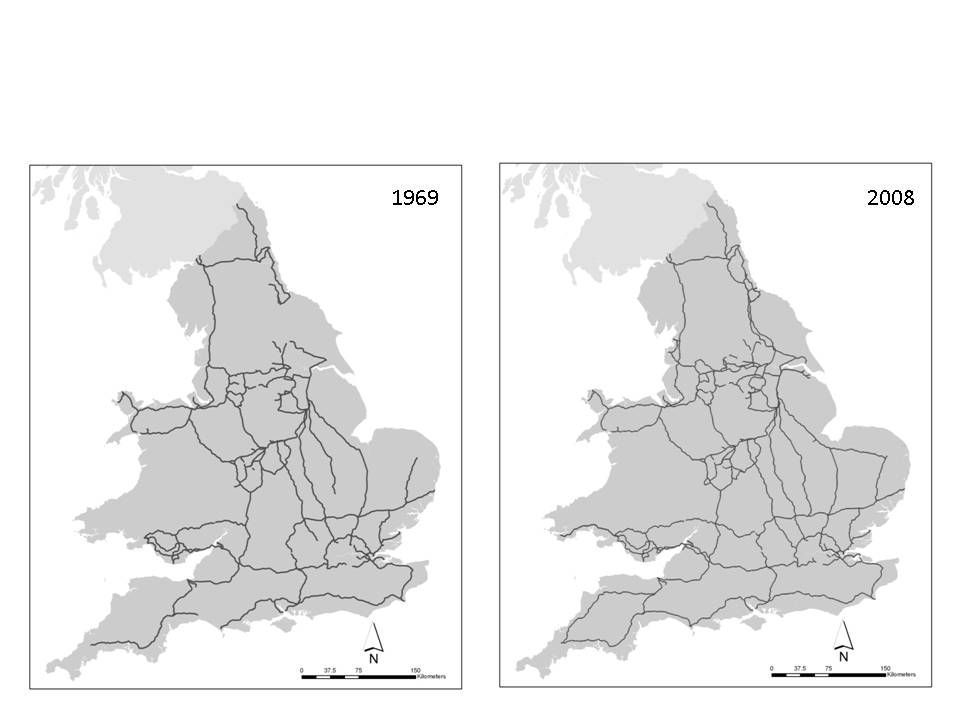


**Supplementary Figure 2. Application of the model to calculate annual mean *net air ion densities* for hypothetical address locations.**


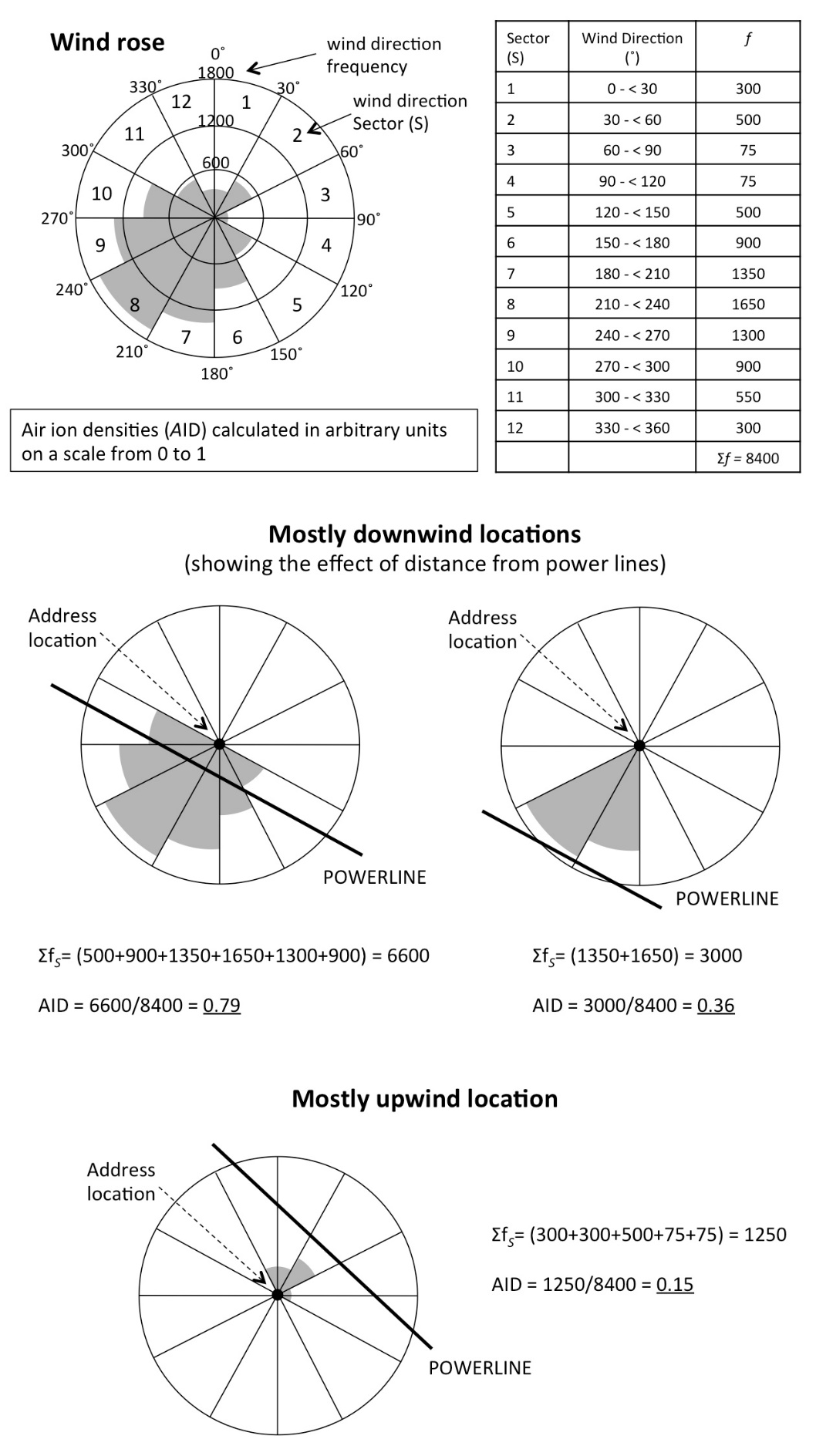

Supplement: dyz275_Supplementary_Data [file dyz275_supplementary_data.zip › dyz275-Suppl_Data/ije-2019-06-0783-File005.docx]
